# The Species Identification in Traditional Herbal Patent Medicine, Wuhu San, Based on Shotgun Metabarcoding

**DOI:** 10.3389/fphar.2021.607200

**Published:** 2021-02-16

**Authors:** Jinxin Liu, Weishan Mu, Mengmeng Shi, Qing Zhao, Weijun Kong, Hongbo Xie, Linchun Shi

**Affiliations:** ^1^ Hebei Key Laboratory of Study and Exploitation of Chinese Medicine, Chengde Medical University, Chengde, China; ^2^ Institute of Medicinal Plant Development, Chinese Academy of Medical Sciences, Peking Union Medical College, Beijing, China

**Keywords:** Wuhu San, shotgun metabarcoding, DNA barcoding, traditional herbal patent medicine, species identification

## Abstract

Traditional herbal patent medicine typically consists of multiple ingredients, making it challenging to supervise contamination by impurities and the improper use of raw materials. This study employed shotgun metabarcoding for the species identification of biological ingredients in traditional herbal patent medicine, Wuhu San. The five prescribed herbal materials found in Wuhu San were collected, and their reference sequences were obtained by traditional DNA barcoding using Sanger sequencing. Two lab-made and three commercial Wuhu San samples were collected, and a total of 37.14 Gb of shotgun sequencing data was obtained for these five samples using the Illumina sequencing platform. A total of 1,421,013 paired-end reads were enriched for the Internal Transcribed Spacer 2 (ITS2), *psbA* and *trnH* intergenic spacer region (*psbA-trnH*), maturase k (*matK*), and ribulose-1, 5-bisphosphate carboxylase (*rbcL*) regions. Furthermore, 80, 11, 9, and 8 operational taxonomic units were obtained for the ITS2, *psbA-trnH*, *matK*, and *rbcL* regions, respectively, after metagenomic assembly, annotation, and chimeric detection. In the two lab-made mock samples, all labeled ingredients in the Wuhu San prescription were successfully detected, and the positive control, *Panax quinquefolius* L., was detected in the HSZY172 mock sample. Three species, namely *Angelica sinensis* (Oliv.) Diels, *Saposhnikovia divaricata* (Turcz. ex Ledeb.) Schischk., and *Carthamus tinctorius* L., belonging to three labeled ingredients, Angelicae Sinensis Radix (Danggui), Saposhnikoviae Radix (Fangfeng), and Carthami Flos (Honghua), were detected in the three commercial samples. *Angelica dahurica* (Hoffm.) Benth. & Hook. f. ex Franch. & Sav., the original Angelicae Dahuricae Radix (Baizhi) species, was only detected in WHS003. *Arisaema erubescens* (Wall.) Schott, *Arisaema heterophyllum* Blume, or *Arisaema amurense* Maxim., the original Arisaematis Rhizoma (Tiannanxing) species, were not detected in any of the commercial samples, which could be attributed to the fact that this medicinal material underwent extensive processing. In addition, the *Saposhnikovia divaricata* adulterant was detected in all the commercial samples*,* while 24 fungal genera, including *Aspergillus*, were identified in both the lab-made and commercial samples. This study showed that shotgun metabarcoding provided alternative strategy and technical means for identifying prescribed ingredients in traditional herbal patent medicine and displayed the potential to effectively complement traditional methods.

## Introduction

In recent years, traditional herbal medicine has been widely used to prevent and treat clinical diseases. Many countries have been using traditional herbs to prevent disease or improve health to varying degrees (Barnes et al., 2016; Job et al., 2016; Sammons et al., 2016; Teng et al., 2016). It is difficult to identify the specific content of traditional herbal patent medicines since they mostly consist of multiple mixed ingredients. Microscopic and physicochemical identification are currently the primary methods used for quality control and the determination of traditional herbal patent medicine content (Committee, 2020). However, the microscopic characteristics of the medicinal materials within multiple original plants may be inconsistent (Chen et al., 1998). Furthermore, insufficient professional talent has also restricted the development of microscopic identification. Physical and chemical identification is based on chemical properties. However, the chemical composition of traditional herbal patent medicine is complex, and the correspondence between the chemical composition and different prescription ingredients may not be clear. In addition, many factors, such as the original plant, environment, harvesting, and processing, may affect the content of active ingredients. It is also possible that some manufacturers illegally add chemical substances, complicating the quality control of traditional herbal patent medicine via chemical composition detection (Xu et al., 2014; Li et al., 2015b). With the development of high-throughput sequencing (HTS) technology, shotgun metagenomics based on the genetic information of species has been successfully applied to identify ingredients in mixed samples. This technique involves the untargeted sequencing of all biological ingredient genomes present in a sample (Quince et al., 2017) to break the metagenomic DNA into small fragments, after which bioinformatics methods are used for assembly without the need for PCR amplification. Therefore, potential biases caused during PCR amplification can be eliminated, and multiple DNA barcodes can be obtained simultaneously for further study to produce more comprehensive data. If the information is used to analyze the traditional DNA barcode region, it is known as shotgun metabarcoding. This was applied here for the species identification of the biological ingredients in traditional herbal patent medicine. Furthermore, shotgun metabarcoding can be a powerful supplement to the conventional identification method used for traditional herbal patent medicine.

Currently, shotgun sequencing technology is primarily used in microbiology to study the composition and functions of microbial communities in different environment samples (Tringe et al., 2005; Warden et al., 2016). A comprehensive study of the microbiome during different processing steps in the beef production chain revealed that the relative abundance of common pathogenic and non-pathogenic bacteria decreased significantly in the final stage, while the relative abundance of some bacteria or pathogens increased. The study proved that shotgun sequencing technology could be used to evaluate the microbial community composition during beef production, as well as pathogen population shifts (Yang et al., 2016). Several studies have shown that shotgun sequencing technology is also applicable to the study of microbial communities in food or beverages that require fermentation (Ferrocino et al., 2018; Arikan et al., 2020), as well as human microbes found in the skin (Oh et al., 2014), saliva (Hasan et al., 2014), and gastrointestinal tract (Vangay et al., 2018; Zhao et al., 2018). In addition to research in the field of microbiology, shotgun sequencing technology has also been successfully used in animal diet analysis (Srivathsan et al., 2015), animal diversity (Zhou et al., 2013), and ingredient identification in food (Haiminen et al., 2019). The development of high-throughput sequencing technology has allowed the application of research strategies based on DNA barcodes to identify traditional herbal patent medicine. A previous study used high-throughput sequencing to identify the biological components in Yimu Wan, a traditional patent medicine. The results showed that all the prescription ingredients could be detected based on the ITS2 sequences, indicating that this technique can be used effectively to detect the legality and safety of Yimu Wan (Jia et al., 2017). Another study used single-molecule, real-time sequencing to identify multiple ingredients in Jiuwei Qianghuo Wan, and the result showed that seven prescription ingredients and positive controls were successfully detected in the two reference samples. Adulterants and potential contaminant species were also found in the commercial samples, indicating that this method can effectively detect the biological components of Chinese patent medicines (Xin et al., 2018b). Furthermore, a study based on high-throughput sequencing and ITS2 regions detected some prescription ingredient adulterants (Cangzhu and Tiannanxing) in traditional Ruyi Jinhuang San medicine (Shi et al., 2018). However, minimal studies are available involving the species identification in traditional herbal patent medicine based on shotgun sequencing. Xin et al. reported the first systematic study involving species detection in traditional herbal patent medicine based on shotgun sequencing (Xin et al., 2018a). The results showed that the ITS2 region could detect all the prescription ingredients, as well as the positive control in the mock samples of Longdan Xiegan Wan. This confirms that shotgun metagenomic sequencing can be used to identify the biological ingredients in traditional herbal patent medicine.

Wuhu San was first recorded in the book, *Si He Ting Ji Fang*, written by Ling Huan during the Qing Dynasty (Deng et al., 2000). It is a type of powder patent medicine prepared by mixing five Chinese medicinal materials, namely Angelicae Sinensis Radix (Danggui), Saposhnikoviae Radix (Fangfeng), Angelicae Dahuricae Radix (Baizhi), Carthami Flos (Honghua), and Arisaematis Rhizoma Preparatum (Zhitiannanxing). The corresponding Latin names of the original species are shown in Supplementary Table S1. It promotes blood circulation, relieves pain, reduces swelling, and disperses blood stasis. Not only can it be used externally with white wine, but it can also be administered with warm yellow rice wine or warm, boiled water. Pharmacological studies have shown that Wuhu San has a relatively apparent anticoagulant effect (Jia et al., 1997), while its alcohol extract displays excellent anti-inflammatory and analgesic properties (Yang et al., 1990). Although both the active substances in Angelicae Sinensis Radix (Danggui) and Carthami Flos (Honghua) have an excellent inhibitory effect on platelet aggregation and can improve blood flow (Li et al., 2009; Zhang et al., 2009), combining these two medicinal materials is more effective in promoting blood circulation (Yue et al., 2017). Moreover, prescription ingredients, such as Saposhnikoviae Radix (Fangfeng), Angelicae Dahuricae Radix (Baizhi), and Arisaematis Rhizoma (Tiannanxing) display certain anti-inflammatory and analgesic properties (Okuyama et al., 2001; Kang et al., 2008; Chunna et al., 2015).

This study uses Wuhu San as an example to evaluate the feasibility and efficacy of using shotgun metabarcoding to identify the biological ingredients in traditional herbal patent medicine. Two mock samples were prepared according to the official species composition listed in the 2015 edition of the Chinese Pharmacopeia and used to verify the feasibility of the shotgun metabarcoding method. This technique was then employed to determine the biological species composition in the commercial Wuhu San samples, aiming to provide different strategies and technical means for the prescription ingredient identification and quality control of traditional herbal patent medicine, such as Wuhu San.

## Materials and Methods

### Herbal Material, Lab-Made Mock Wuhu San, and Commercial Wuhu San Samples

**FIGURE 1:**
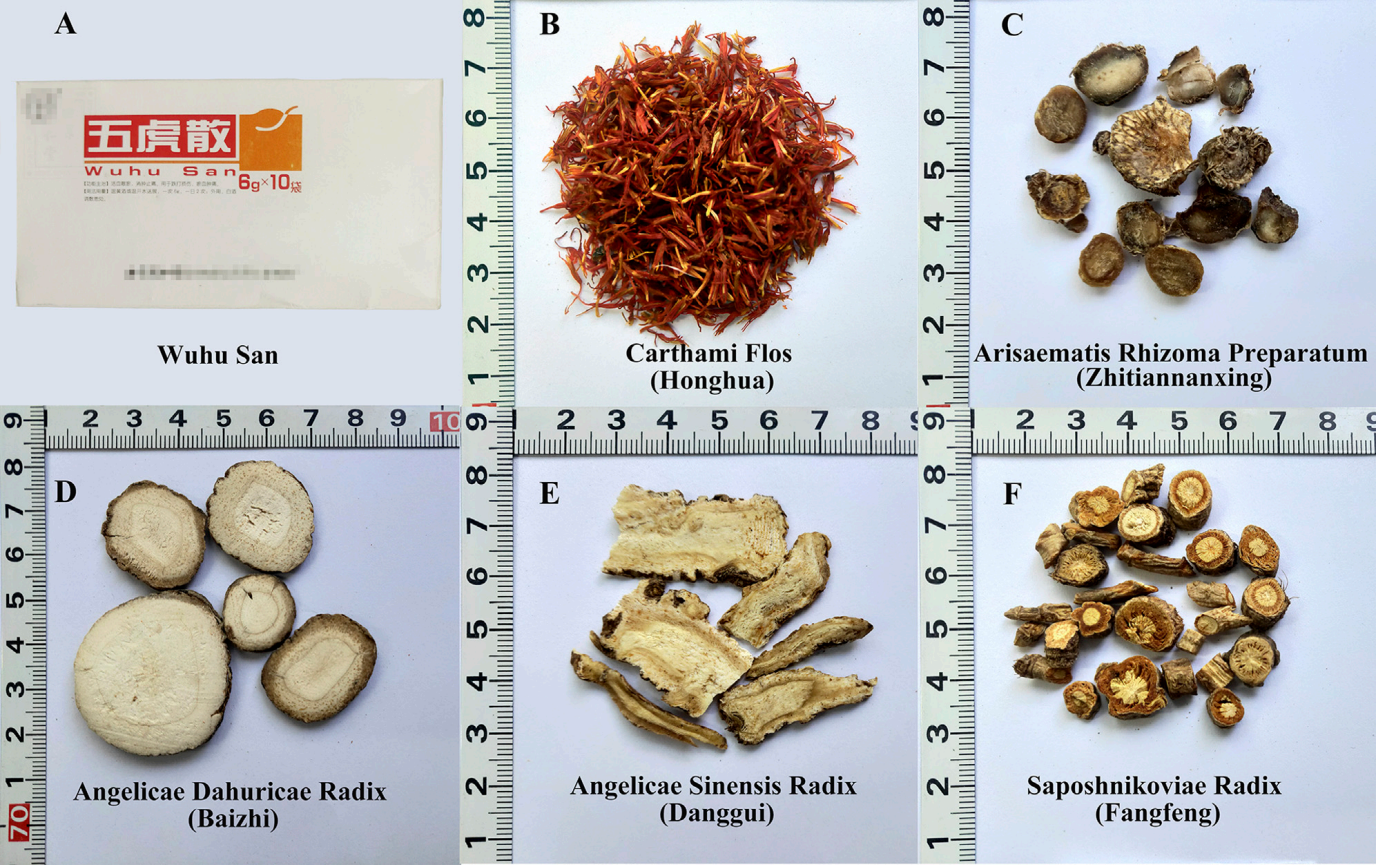
The morphological characteristics of five herbal materials in the prescription of Wuhu San **(A)** Wuhu San **(B)** Carthami Flos (Honghua) **(C)** Arisaematis Rhizoma Preparatum (Zhitiannanxing) **(D)** Angelicae Dahuricae Radix (Baizhi) **(E)** Angelicae Sinensis Radix (Danggui), and **(F)** Saposhnikoviae Radix (Fangfeng).

Four kinds of Wuhu San herbal materials, namely Angelicae Sinensis Radix (Danggui), Saposhnikoviae Radix (Fangfeng), Angelicae Dahuricae Radix (Baizhi), Carthami Flos (Honghua), were collected from the Beijing TRT pharmaceutical company. Arisaematis Rhizoma (Tiannanxing) was obtained from Chengde, Hebei Province (Supplementary Table S2 and Figure 1). The herbal materials were authenticated using the morphological and traditional DNA barcoding methods. The lab-made mock samples were prepared according to the prescription ingredients and manufacturing method of Wuhu San listed in the 2015 edition of the Chinese Pharmacopoeia (Table 1). Of these, Arisaematis Rhizoma (Tiannanxing) was processed in advance following the method described in the Chinese Pharmacopoeia for Arisaematis Rhizoma Preparatum (Zhitiannanxing). The two lab-made mock samples were prepared and labeled as HSZY160 and HSZY172. The Panacis Quinquefolii Radix (Xiyangshen) powder was added to HSZY172 as a positive control at an amount equal to Angelicae Dahuricae Radix (Baizhi), representing the lowest herbal ingredient in the Wuhu San prescription. In addition, the three commercial Wuhu San samples were acquired from pharmacies and labeled WHS001, WHS002, and WHS003.

**TABLE 1:** The proportion of the five ingredients listed in the prescription of Wuhu San.

Pinyin name	Latin name	Proportion in the preparation (%)
Danggui	Angelicae Sinensis Radix	21.3
Honghua	Carthami Flos	21.3
Fangfeng	Saposhnikoviae Radix	21.3
Zhitiannanxing	Arisaematis Rhizoma Preparatum	21.3
Baizhi	Angelicae Dahuricae Radix	14.6

### DNA Extraction, PCR Amplification, Sanger Sequencing, and HTS

The DNA extraction of the herbal material samples was performed according to previous research (Liu et al., 2017) and the DNA barcoding principles for traditional Chinese herbal medicine (Chen et al., 2014) using a plant genomic DNA extraction kit (Tiangen Biochemical Technology (Beijing) Co., Ltd., China). The meta-genomic DNA of Wuhu San was extracted according to the previously published protocols of the CTAB-based method (Cheng et al., 2015) with some changes. A pre-wash buffer was used for pretreatment (Xin et al., 2018a), after which lysis buffer was added. The samples were placed in a 56°C water bath overnight for lysis. Extraction was performed using chloroform/isoamyl alcohol (volume ratio 24:1), and phenol/chloroform/isoamyl alcohol (volume ratio 25:24:1). The DNA was purified by adding 50 μL of sodium acetate and 1,250 μL of 100% methanol. The extracted DNA quality was estimated using a NanoDrop one ultra-micro spectrophotometer (Thermo Fisher Scientific Inc., USA). The traditional DNA barcoding regions of ITS2, *psbA-trnH*, *matK*, and *rbcL* were amplified with DNA barcoding primer sets and conditions proposed by the barcodes of the traditional Chinese herbal medicine data system (TCM-BOL) (Chen et al., 2014), the CBOL plant working group (Group et al., 2009), and the barcode of life data system (BOLD) (Ratnasingham and Hebert, 2007) using 2 × Taq master mix (AidLab Biotechnologies Co., Ltd., China). The PCR products were bi-directionally sequenced on an ABI 3730xL DNA Analyzer (Thermofisher Co., Ltd., United States). After constructing a PCR-free library, the Wuhu San DNA was sheared into fragments and sequenced using the Illumina NovaSeq platform.

### Data Analysis

The Sanger sequencing results were obtained according to the *Standard DNA barcodes of Chinese Materia Medica in Chinese Pharmacopoeia* edited by Chen Shilin (Chen, 2015). The sequence chromatograms were assembled, and the primers were removed using Codoncode aligner v 9.0.1 (CodonCode Corp., Dedham, MA, United States). For the Illumina sequencing data, the sequencing adapter and low-quality reads were filtered using Trimmomatic v0.38 (Bolger et al., 2014). The paired-end reads were enriched using local python scripts (Shi et al., 2019). The enriched reads belonging to ITS2, *psbA-trnH*, *matK,* and *rbcL* were assembled using MEGAHIT v1.2.9 and MetaSPAdes v3.13.2 (Li et al., 2015a; Nurk et al., 2017). Contigs obtained via the two types of software were merged, and duplicates were removed with cd-hit at 100% identity (Li and Godzik, 2006). The ITS2 regions were obtained using the hidden Markov model (HMM)-based annotation method (Keller et al., 2009). The traditional DNA barcoding regions of *psbA-trnH*, *matK,* and *rbcL* were acquired by removing primer sequences based on Cutadapt v2.10 (Kechin et al., 2017). The chimera detection of the annotated contigs was performed using UCHIME v4.2 (Edgar et al., 2011). Sequences belonging to each marker were clustered into OTUs at 100% identity using Usearch v11 (https://www.drive5.com/usearch/), and the representative sequence for each OTU was selected for further analysis. The shotgun paired-end reads were mapped to the OTU representative sequences using bowtie2 v2.4.1 (Langmead and Salzberg, 2012), while the sequencing depth and coverage values were calculated using Samtools v1.10 (Etherington et al., 2015). Poor-quality OTUs were removed when its representative sequences displayed a sequencing depth ≤3 or coverage ≤95%. The remaining high-quality OTUs were used for species assignment by searching the TCM-BOL (Chen et al., 2014), BOLD (Ratnasingham and Hebert, 2007), and GenBank (Benson et al., 2018) databases using the basic local alignment search tool, BLAST (Camacho et al., 2009). Finally, the statistics and taxonomic visualization of the species composition of the traditional herbal patent medicine were performed using MEGAN v6.18.9 (Huson et al., 2016).

After the species in the traditional herbal patent medicine were identified via DNA barcodes, some terms related to the identified species were defined as follows:

#### Authentic

The species in the medicinal materials are authentic if it is identified as one of the labeled ingredients in the prescription of the traditional herbal patent medicine.

#### Substitution

Substitutions refer to the species in the medicinal materials with similar characteristics such as efficacy, chemical composition, pharmacological effect, and clinical effect, which are selected instead of authentic medicinal materials according to the clinical medication plan when there is a shortage of these materials (Tang and Huang, 1994; Suo and Chen, 2006).

#### Adulterant

Adulterants refer to the species in the medicinal materials that are used as authentic although they are similar in appearance or have the same name as the authentic material, but are different regarding the original plant source, chemical composition, pharmacological effect, and clinical effect (Tang and Huang, 1994).

#### Contaminant

Contaminants include fungal contamination and impurities.

## Results

### The Authentication of the Five Herbal Materials in Wuhu San and Their ITS2, *psbA-trnH*, *matK*, and *rbcL* DNA Barcodes

The five herbal materials labeled on Wuhu San prescription were collected from Chengde (Hebei province). They were first identified using the morphological method and then authenticated using DNA barcoding to ensure the accuracy of the mock samples. High-quality DNA was extracted from these materials, after which the ITS2, *psbA-trnH*, *matK*, and *rbcL* DNA barcodes were amplified using their corresponding universal primers. Except for the *psbA-trnH* sequence of Arisaematis Rhizoma (Tiannanxing) and the *rbcL* sequence of Angelicae Dahuricae Radix (Baizhi), all the ITS2, *psbA-trnH*, *matK*, and *rbcL* DNA barcodes of the five herbal materials were successfully amplified and then bi-directionally sequenced using Sanger sequencing technology. The GenBank accession numbers of these sequences are shown in Table 2. The ITS2 and *psbA-trnH* sequences obtained via Sanger sequencing were assigned to species by blasting to the TCM–BOL system, while the *matK* and *rbcL* DNA barcodes obtained using the same method were assigned to a species or genus using the BOLD system and GenBank NT database. By combining the identification results of the four DNA barcodes, all five herbal materials were authenticated, and their original Angelicae Sinensis Radix (Danggui), Saposhnikoviae Radix (Fangfeng), Angelicae Dahuricae Radix (Baizhi), Carthami Flos (Honghua), and Arisaematis Rhizoma Preparatum (Zhitiannanxing) species were assigned to *Angelica sinensis* (Oliv.) Diels, *Saposhnikovia divaricata* (Turcz. ex Ledeb.) Schischk., *Carthamus tinctorius* L., *Angelica dahurica* (Hoffm.) Benth. & Hook. f. ex Franch. & Sav., and *Arisaema amurense* Maxim., respectively.

**TABLE 2:** The GenBank accession numbers of the five herbal materials in Wuhu San and the positive control, Panacis Quinquefolii Radix (Xiyangshen).

Herb medicinal material	ITS2	*psbA-trnH*	*matK*	*rbcL*
Angelicae Sinensis Radix (Danggui)	MN727081	MT994327	MN729559	MN746764
Carthami Flos (Honghua)	MN727076	MT994328	MN729561	MN746766
Saposhnikoviae Radix (Fangfeng)	MT821449	MT994331	MW000338	MW000334
Arisaematis Rhizoma (Tiannanxing)	MT821451	—	MW000340	MW000335
Angelicae Dahuricae Radix (Baizhi)	MT821450	MT994330	MW000339	—
Panacis Quinquefolii Radix (Xiyangshen)	MT102865	MT994329	MW000341	MW000333

Note: "—" indicates that the sequence was not successfully obtained.

### HTS and Shotgun Metabarcoding Data Assembly

The average DNA concentration of the lab-made mock samples and commercial samples was 144.06 ng/μL, while the *A*_*260*_ */A*_*280*_ ranged between 1.8 and 2.0 (Supplementary Table S3). This indicated that the concentration and purity of the DNA extracted from the traditional herbal patent medicine samples were high. A total of 37.14 G of raw data was obtained via HTS, while 8.54 G and 9 G of raw data were acquired from the HSZY160 and HSZY172 lab-made mock samples, respectively. Additionally, 6.81 G, 6.78 G, and 6.01 G of raw sequencing data were acquired from the WHS001, WHS002, and WHS003 commercial samples. A total of 123,799,141 paired-end sequencing reads were obtained. After removing low-quality sequences, a total of 1,421 013 paired-end sequencing reads were enriched for the ITS2, *psbA-trnH*, *matK*, and *rbcL* regions. The detailed sequencing results are shown in Supplementary Table S4. A total of 6,884 unique contigs were generated by assembling and then removing duplications using MEGAHIT v1.2.9 and MetaSPAdes v3.13.2. The DNA barcoding regions of ITS2, *psbA-trnH*, *matK*, and *rbcL* yielded 136, 26, 16, and 21 unique contigs, respectively, after annotating and removing the primers. The cluster analysis of the ITS2 region yielded a total of 80 OTUs, with an average length of 207.5 bp and an average GC content of 54.4%. The number of OTUs obtained via nuclear ITS2 was more than seven times that of chloroplast *psbA-trnH*, *matK*, and *rbcL*. Moreover, the GC content of the ITS2 sequences was higher than that of the *psbA-trnH*, *matK*, and *rbcL* sequences*.* The specific data results of the four markers are shown in Table 3.

**TABLE 3:** The results of the data analysis of four marker types.

	ITS2	*psbA-trnH*	*matK*	*rbcL*
Number of unique contig	378	6,416	44	46
Number of DNA barcodes after annotation chimera	136	26	16	21
Number of OTU	80	11	9	8
Average length (bp)	207.5	321.1	836.3	703
GC content (%)	54.4	32.2	34.7	44.1

### The Accuracy Verification of the DNA Barcoding Sequences Assembled Using the Shotgun Sequencing Data of the Lab-Made Samples

The DNA barcode assembly results of the labeled ingredients in the prescription of the lab-made mock samples are shown in Table 4. To determine the assembly accuracy of the DNA barcode regions assembled via shotgun sequencing, the sequences obtained using shotgun metabarcoding and the reference sequences of ITS2, *psbA-trnH*, *matK*, *rbcL* obtained via Sanger sequencing were analyzed for consistency. The *psbA-trnH* sequence of *Arisaema amurense* and the *rbcL* sequence of *Angelica dahurica* were not obtained with Sanger sequencing.

**TABLE 4:** The DNA barcode sequences of five ingredients in the prescription of the lab-made Wuhu San samples, and the positive control, *Panax quinquefolius,* obtained via shotgun metabarcoding.

Species	HSZY160				HSZY172			
	ITS2	*psbA-trnH*	*matK*	*rbcL*	ITS2	*psbA-trnH*	*matK*	*rbcL*
*Angelica sinensis*	√	√	√	√	√	√	√	√
*Carthamus tinctorius*	√	√	√	√	√	√	√	√
*Saposhnikovia divaricata*	√	√	√	√	√	√	√	√
*Arisaema amurense*	√	—	√	√	√	—	√	√
*Angelica dahurica*	√	√	—	—	√	√	—	—
*Panax quinquefolius*					√	√	√	√

Note: "√" shows that the corresponding assembly sequence of this species was obtained; "—" indicates that the corresponding assembly sequence of this species cannot be obtained.

Regarding the ITS2 sequences, the assembly sequences of all the ingredients in the prescriptions of the two mock samples were obtained. The sequence bases of *Angelica sinensis* and *Angelica dahurica* were identical to their reference sequences. A one base difference was evident between the sequences of *Arisaema amurense* obtained via two sequencing methods. Two *Saposhnikovia divaricata* assembly sequences were obtained, one of which was identical to the reference sequence bases, while the other differed by three bases. Two *Carthamus tinctorius* assembly sequences were obtained, which differed from the reference sequence by 0 and one base, respectively.

For the *psbA-trnH* sequences, the assembly sequences of *Angelica sinensis*, *Saposhnikovia divaricata*, *Carthamus tinctorius*, and *Angelica dahurica* were successfully obtained, but shotgun metabarcoding failed to acquire the *Arisaema amurense* sequence. The assembled sequences of *Angelica sinensis*, *Angelica dahurica*, and *Carthamus tinctorius* were identical to the reference sequences obtained via Sanger sequencing. Two *psbA-trnH* sequences of *Saposhnikovia divaricata* were acquired via shotgun sequencing. Compared with the sequences obtained via traditional DNA barcoding, one is identical, while the other displays two base differences.

Regarding the *matK* sequences, the assembly sequences were acquired for all the species in the two mock samples except for *Angelica dahurica*. A comparison between the *matK* assembly and reference sequences of the four species showed that the sequence bases of *Arisaema amurense* were the same. The two assembled sequences of *Carthamus tinctorius* and *Saposhnikovia divaricata* were obtained via shotgun sequencing and displayed a 0–3 base difference from the reference sequences. A total of two assembled *matK* sequences of *Angelica sinensis* were obtained, which differed from the reference sequences by five and seven bases, respectively.

The assembly sequences of *Carthamus tinctorius* and *Arisaema amurense* were successfully acquired for the *rbcL* region. There were only five mutation sites among the *rbcL* sequences of *Angelica sinensis*, *Saposhnikovia divaricata,* and *Angelica dahurica*. Therefore, these sequences of the three species could not be assembled separately. Only the *rbcL* sequences of *Carthamus tinctorius* and *Arisaema amurense* were obtained using both shotgun and Sanger sequencing. The assembled sequences of the two species were completely consistent with the reference sequences.

In addition, for the positive control, *Panax quinquefolius,* all the ITS2, *psbA-trnH*, *matK*, and *rbcL* sequences that were acquired using the shotgun metabarcoding method were identical to those obtained via the traditional DNA barcode method.

### The Plant Species Composition of Commercial Wuhu San Samples Identified Through Shotgun Metabarcoding

**FIGURE 2:**
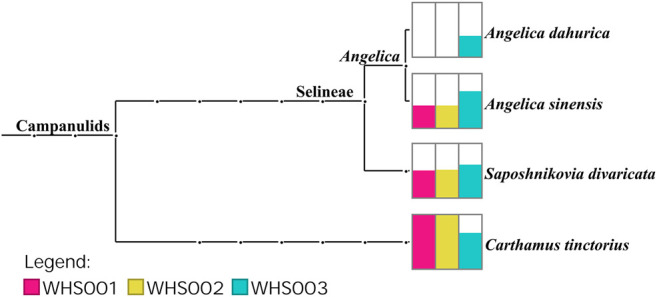
Taxonomic analysis of three samples detected via reads belonging to the ITS2 region. Each taxonomic node is drawn as a bar chart indicating the number of reads assigned to the taxon for each sample.

**FIGURE 3:**
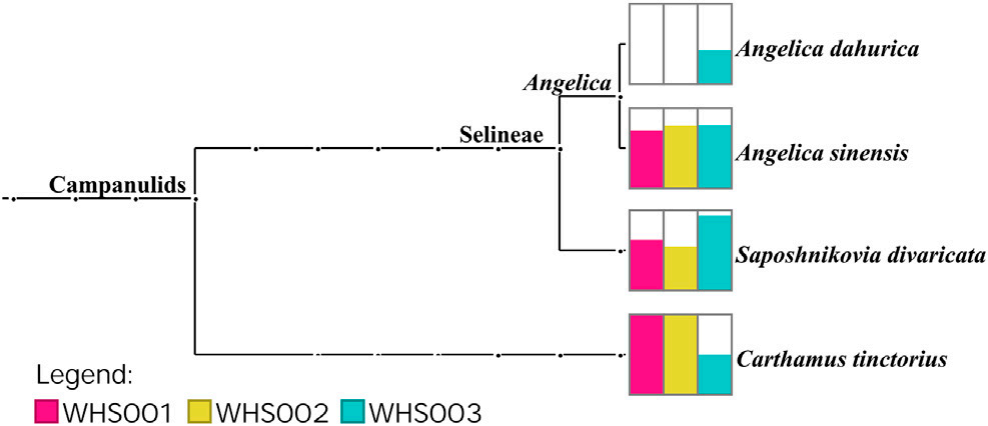
The taxonomic analysis of three samples detected by reads belonging to the *psbA-trnH* region. Each taxonomic node is drawn as a bar chart indicating the number of reads assigned to the taxon for each sample.

Regarding the labeled ingredients in the prescription, combined with the ITS2, *psbA-trnH*, *matK*, and *rbcL* regions, the three commercial samples contained prescription medicinal materials *Angelica sinensis*, *Carthamus tinctorius*, and *Saposhnikovia divaricata*. None of the samples contained *Arisaema erubescens*, *Arisaema heterophyllum*, or *Arisaema amurense*, representing the original Arisaematis Rhizoma (Tiannanxing) species, while only *Angelica dahurica* was detected in WHS003 (Figures 2, 3, Supplementary Figures S1, S2). For the ITS2 and *psbA-trnH* sequences*,* the results showed that *Angelica sinensis* and *Saposhnikovia divaricata* were detected in all three commercial samples, while *Angelica dahurica* was only detected in WHS003 (Figures 2, 3). A total of six OTUs were obtained from the *matK* region. Of these, two OTUs were identified as *Carthamus tinctorius*, while the remaining four were only identified as belonging to the Apiaceae family, but the species could not be authenticated (Supplementary Figure S1). Six OTUs were obtained from the *rbcL* sequences in the three commercial samples, of which one was identified to species level, namely *Carthamus tinctorius*. The remaining five OTUs could only be identified as belonging to the Apiaceae family, but no species could be determined (Supplementary Figure S2). Detailed reads of the prescription ingredients in the three commercial samples based on four barcodes are shown in Supplementary Tables S5–S8.

As for the adulterants of the labeled ingredients, *Ferula bungeana* Kitag. was detected in two of the commercial samples (WHS001 and WHS002) based on the ITS2 sequences. In addition to these labeled ingredients and their adulterants, several other potential impurities were found in the commercial Wuhu San samples. Based on the ITS2 sequences, *Scutellaria baicalensis* Georgi was detected in two commercial samples (WHS001 and WHS002), while *Salix* L. was detected in WHS002 and WHS003. Impurities, such as *Convolvulus arvensis* L., *Chenopodium album* L., and *Citrus* L., were also found in WHS003.

### The Fungal Contamination of the Lab-Made and Commercial Wuhu San Samples Detected via ITS2

A total of 36 fungal OTUs were obtained based on ITS2 sequences, including 22 families and 24 genera. The reads number of the *Rhizopus* genus was the highest of the 24 detected genera, accounting for 94.33% of the total number of fungal reads. It was the predominant genera in the two lab-made mock samples and three commercial samples. Fungi belonging to the *Macrophomina*, *Fusarium*, *Aspergillus,* and *Alternaria* genera were the dominant abundant in this study. Most of these fungi were molds that were present during the storage of herbal medicines, while some were soil habitant fungi.

**FIGURE 4:**
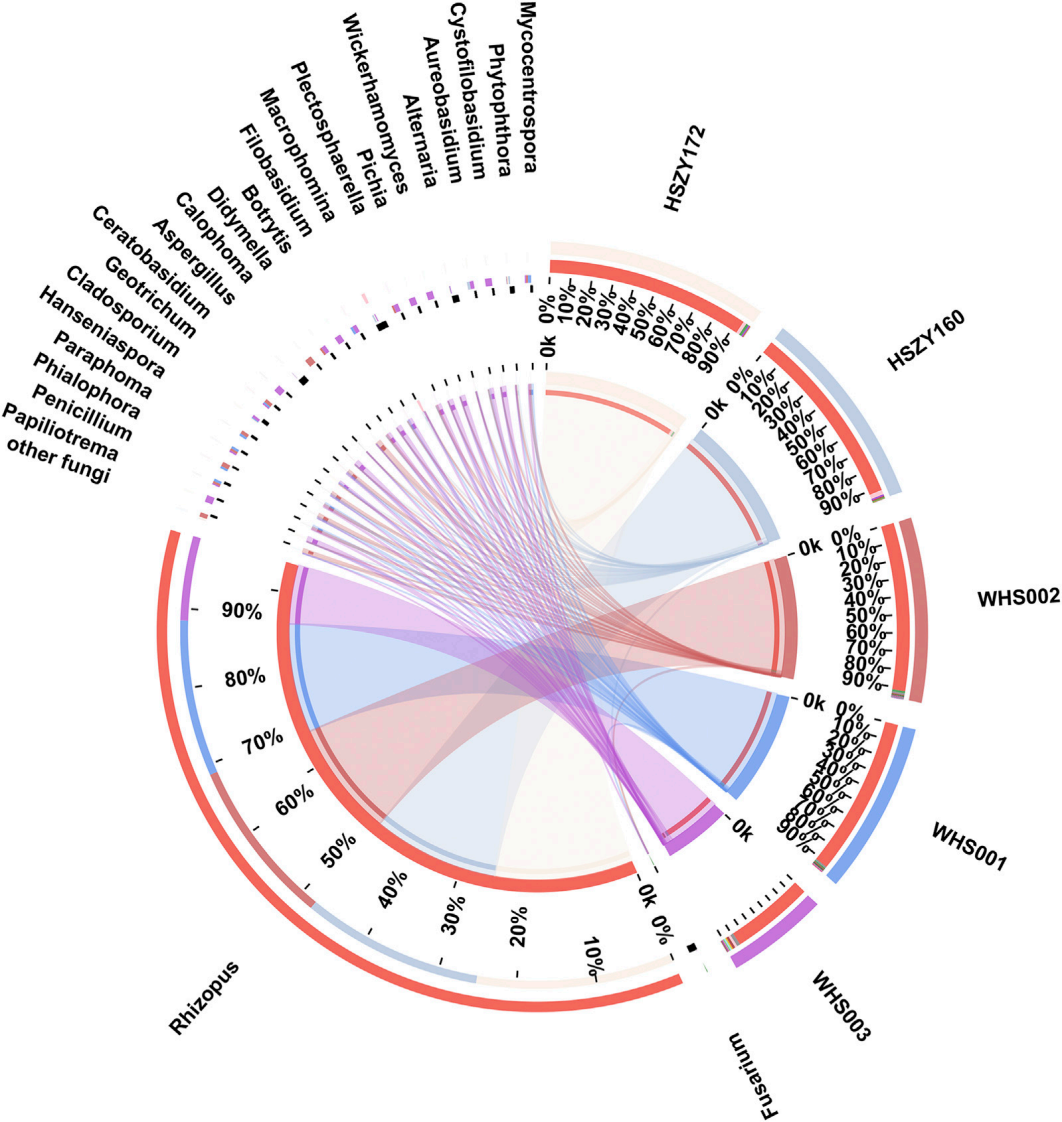
Distribution of the fungi for each sample at the genus level. The data were visualized using Circos. The left half-circle indicates the distribution ratio of species in different samples at the genus level where the outer ribbon represents the species, the inner ribbon represents different groups, and the length represents the sample proportion of a particular genus. The right half-circle indicates the species composition in each sample where the color of the outer ribbon represents samples from different groups, the color of the inner ribbon represents the composition of different species in each sample, and the length of the ribbon represents the relative abundance of the corresponding species.

Here, 8, 7, 13, 16, and 20 fungal genera were detected in HSZY160, HSZY172, WHS001, WHS002, and WHS003, respectively. Fungi belonging to the *Fusarium*, *Rhizopus,* and *Alternaria* genera were detected in all five samples. In addition, the composition of the identified fungi in the two lab-made samples (HSZY160 and HSZY172) was similar at the genus level. The number of fungal species found in the commercial samples was significantly higher than in the mock samples, and some differences were evident between the fungal compositions of the three commercially available samples (Figure 4, Supplementary Tables S9). Besides the fungi belonging to genera found in all five samples, *Aspergillus*, *Penicillium*, *Geotrichum,* and *Mycocentrospora* were detected in all three commercial samples. WHS001 and WHS002 displayed a higher similarity in fungal composition than WHS003. Most of the fungal species were detected in WHS003, while *Aspergillus flavus,* which may produce aflatoxin that is harmful to human health, was detected in this sample (Supplementary Figure S3, Supplementary Tables S10).

## Discussion

### The Feasibility of Shotgun Metabarcoding Technology in Authenticating the Herbal Ingredients of Wuhu San

DNA metabarcoding is currently the most widely used detection method for mixed biological samples. Many studies are available that involve the identification of biological ingredients of traditional herbal medicine based on DNA metabarcoding technology (Jia et al., 2017; Xin et al., 2018b; Shi et al., 2018; Zhang et al., 2020). However, the PCR amplification efficiency of universal DNA barcode primers is affected by the severe DNA degradation of traditional herbal medicine (Xin et al., 2018a). Furthermore, it may result in potential bias during PCR amplification using primers (Berry et al., 2011). Shotgun metabarcoding directly performs library construction and sequencing of the total DNA of mixed samples (Quince et al., 2017) and can obtain ITS2 sequences and multiple chloroplast DNA barcode sequences through the assembly for species identification (Xin et al., 2018a). This method has also been applied for studying clinical or complex environmental samples (Nielsen et al., 2014; Vangay et al., 2018). The shotgun metabarcoding method can reduce or eliminate the potential bias caused by PCR amplification and obtain a longer DNA barcode sequence interval than DNA metabarcoding. Several analytical biodiversity studies based on shotgun sequencing technology have shown that this technique can be used for biodiversity assessment. This method avoids the PCR amplification of particular gene markers to display species richness with high fidelity, while there is a significant correlation between the reads and biomass of most species (Zhou et al., 2013; Bista et al., 2018). However, this technology is more expensive, while the DNA quality requirements are also higher. Furthermore, the related bioinformatics analysis also presents a significant challenge.

This study shows that four barcode sequences can be successfully obtained in most of the medicinal materials through shotgun metabarcoding technology in the lab-made mock samples. The mutual verification between the results obtained via different markers further confirmed the accuracy of the method. The results revealed that the *psbA-trnH* sequence of plants belonging to Araceae presented a low success rate via Sanger sequencing due to the adenine (A) and thymine (T) base content of over 70%, while their *psbA-trnH* sequences were difficult to obtain (Luo et al., 2009). It is speculated that the failure to obtain the *psbA-trnH* sequences of *Arisaema amurense* via shotgun metabarcoding technology is due to sequencing reads not being enriched enough for assembly or assembly failure caused by continuous AT repetition. Furthermore, the *Panax quinquefolius* barcode sequences were obtained in the HSZY172 sample, proving the sensitivity of the method for detecting prescription ingredient species. Arisaematis Rhizoma (Tiannanxing) was not detected in the three commercially available samples through any of the markers. It is speculated that this is due to the difference in the degree of processing of Arisaematis Rhizoma Preparatum (Zhitiannanxing) when preparing the lab-made samples and commercial products. The DNA degradation of Arisaematis Rhizoma Preparatum (Zhitiannanxing) in the commercial samples may be more severe. *Ferula bungeana*, a plant belonging to the Apiaceae, was detected in two of the commercial samples. Studies have shown that the dried roots of the *Ferula bungeana* are used as Saposhnikoviae Radix (Fangfeng) in the market (Yang, 2006; Chen and Chen, 2010). However, the efficacy of the two medicinal materials is quite different, and the identification of the herbal materials should be enhanced to ensure the efficacy of traditional herbal patent medicine. Moreover, herbal impurities, such as *Salix* sp., *Chenopodium album*, *Convolvulus arvensis*, *Citrus* sp. and *Scutellaria baicalensis* were also detected. Their presence may be due to weeds that have been mixed in during harvesting (Geng et al., 2018) or accidental cross-contaminants from the same production line (Xin et al., 2018b). Furthermore, fungi were found in all the samples. During the planting, harvesting, transportation, and storage of herbal medicines, improper methods may cause fungal growth or the accumulation of mycotoxins (Ying et al., 2016), directly affecting the quality, efficacy, and safety of herbal medicines. This necessitates the examination of optimal storage conditions or preparation methods of herbal medicines contaminated by fungi (Wang, 2016). In summary, this indicated that shotgun metabarcoding could not only detect the adulteration of herbal materials in traditional herbal patent medicine, but it can also detect exogenous contamination, such as fungi and impurities. This proves the feasibility of shotgun metabarcoding for detecting biological ingredients in the traditional herbal patent medicine, Wuhu San.

### The Challenges of the Current Shotgun Metabarcoding Method During Data Analysis

#### False Positives Caused by Reads Mapping in Conserved Regions

In this study, the ITS2 sequence of *Peucedanum japonicum* Thunb. was assembled from the lab-made mock samples. The coverage of the reads mapping was 100%, and the sequencing depth was 1,445.61. However, *Peucedanum japonicum* was not added to the lab-made mock samples. Visual reads mapping based on Codoncode Aligner indicated that the tail of the ITS2 sequence had a mapping depth exceeding 2000 ×, but only two mapping reads were present at the front end. Although the coverage of the fragments was uneven, the exceptionally high coverage of the tail significantly increased the overall coverage of the sequence, leading to the occurrence of false-positive sequences. Intercepting the tail segment, the NCBI BLAST analysis indicated that it was a 28S conserved sequence (Supplementary Figure S4). Further investigation revealed that the ITS2 sequence tail assembly was not accurate, preventing the ITS2 sequence annotation process from recognizing the 28S section, partially cutting it off. The 28S region is exceptionally conservative. Bowtie2 software randomly maps the reads to the reference genome with the same sequences during the mapping process (Langmead and Salzberg, 2012), resulting in an extremely high mapping depth for the conservative 28S region and a high average sequencing depth for the ITS2 sequences. This problem highlights the necessity to perform sequence annotation and primer removal accurately. Furthermore, CodonCode Aligner software can also verify the annotation results to reduce the occurrence of false-positive sequences.

#### The Accuracy of High Similarity Sequence Assembly

The assembly of high similarity sequences or low variability sequences is a challenge during shotgun metabarcoding data analysis (Quince et al., 2017). The optimized metagenomic data assembly software and more extensive k-mer parameters may overcome the assembly errors of lower similarity sequences to some extent. However, some difficulties remain when assembling the *matK* and *rbcL* sequences of some species in the same family, especially in the same genus. In this study, the ITS2 and *psbA-trnH* sequences of *Angelica dahurica* were obtained from the lab-made mock samples and commercial samples, but the *matK* and *rbcL* sequences of *Angelica dahurica* were not assembled. The prescriptions of Wuhu San contain *Angelica dahurica*, *Angelica sinensis,* and *Saposhnikovia divaricata* of the same family. The *matK* and *rbcL* sequences of the three species exhibited a similarity of more than 98%. It is speculated that the assembly of the three species may be incorrect due to insufficient assembly accuracy. The *matK* sequences of *Angelica dahurica*, *Angelica sinensis,* and *Saposhnikovia divaricata* obtained via Sanger sequencing were compared. There were six base differences between *Angelica dahurica* and *Saposhnikovia divaricata*, and 12 base differences between *Angelica dahurica* and *Angelica sinensis*. Furthermore, 14 base differences were evident between the *matK* sequences of *Saposhnikovia divaricata* and *Angelica sinensis* (Supplementary Figure S5). The *matK* sequence of *Angelica dahurica* displayed a higher similarity to that of *Saposhnikovia divaricata*, and differences were apparent in the bases at sites 42, 376, 415, 588, 722, and 758, indicating an average of 139 bp in a variant site. Analysis of the visual reads mapping results based on Codoncode Aligner showed that the base sites mentioned above have specific bases representing *Angelica dahurica* and *Saposhnikovia divaricata,* respectively (Supplementary Figure S6). Therefore, it is inferred that the assembly has not reached a high level of accuracy due to the small difference in sequence bases. The *matK* sequences of two species may be assembled into one sequence, representing the species sequence with more extensive sequencing depth. This is the same in the case of the *rbcL* sequence. Analysis performed via Sanger sequencing revealed that the bases of the *rbcL* sequences of *Angelica dahurica* and *Saposhnikovia divaricata* were T and A at the 270 base site, T and C at the 130 base site, and T and G at the 635 base site, respectively. The bases of the *rbcL* sequences of *Angelica dahurica* and *Angelica sinensis* at base sites 237, 270, and 475 were G/A, T/A, and C/T, respectively (Supplementary Figure S7). The base differences between the *rbcL* sequences of the three species were smaller. There were only five mutation sites among the *rbcL* sequences of *Angelica dahurica*, *Angelica sinensis,* and *Saposhnikovia divaricata*, that is, one variant site appeared on average 141 bp. The *rbcL* sequence mapping results of *Angelica dahurica* showed that different bases represented these three species at the various base positions (Supplementary Figure S8). The average length of a variation site in *matK* and *rbcL* sequences exceeds the length of commonly used k-mer (Quince et al., 2017). Using a more extended k-mer parameter may solve the problem of species distinction when the sequence similarity exceeds 98%. However, the k-mer length may exceed the standard analysis length, requiring a massively distributed metagenome assembler, such as Ray, for *de novo* assembly to solve the computational time and memory challenge (Boisvert et al., 2012).

#### The Identity Threshold of DNA Barcodes for Constructing OTUs

This study initially conducted OTU sequence clustering according to 99% similarity to improve the efficiency and accuracy of the analysis. The results showed that *Angelica sinensis* was detected based on the ITS2, *psbA-trnH,* and *matK* regions, while the *Angelica sinensis* sequence was not detected based on the *rbcL* region. However, the *rbcL* sequence of *Saposhnikovia divaricata,* which belongs to the same family as *Angelica sinensis,* was detected. The Codoncode Aligner was used to further analyze the *rbcL* sequences of *Angelica sinensis* and *Saposhnikovia divaricata* obtained via Sanger sequencing. The results revealed that the *rbcL* sequences of the two species only displayed a 4-base difference. When the similarity was set to 99% for OTU clustering, the *rbcL* sequences of *Angelica sinensis* and *Saposhnikovia divaricata* was artificially divided into an OTU cluster. Therefore, the assembled *rbcL* sequences obtained via shotgun sequencing and the *rbcL* sequences of *Angelica sinensis* and *Saposhnikovia divaricata* acquired with Sanger sequencing were re-analyzed by building phylogenetic trees. Two sequences, namely "WHS001_rbcL_0189_k141_7" and "WHS002_rbcL_0047_k141_8" were found and grouped with the *rbcL* sequences of *Angelica sinensis* (HSYC2002 and HSYC2022) (Supplementary Figure S9). Therefore, the *Angelica sinensis* sequence could be detected by the *rbcL* region. This study further revealed that the *Angelica dahurica*, *Angelica sinensis,* and *Saposhnikovia divaricata* sequences were similar, especially those of *matK* and *rbcL*. Therefore, different levels of similarity should be set for OTU clustering when using DNA barcodes with varying species resolutions. For homologous species, the similarity should be further adjusted to 100% to avoid the undetectable phenomenon of sequences with similarities that are too high, which is also consistent with the current analysis strategy recommended by USEARCH.

### The Species-Discriminating Power of the ITS2, *psbA-trnH*, *matK*, and *rbcL* DNA Barcodes

The four DNA barcodes displayed differences in the species discriminating power for the ingredients of Wuhu San prescriptions. All the ITS2 sequences obtained in this study can accurately identify species after BLAST. However, the *psbA-trnH* sequences of *Panax quinquefolius* and *Panax ginseng* did not have a variable site and could not be accurately distinguished. The regions of *rbcL* and *matK* exhibited certain limitations in identifying Apiaceae species in this study. Another study indicated that the efficiency of *rbcL* and *matK* sequences in identifying Apiaceae species was much lower than that of ITS2 sequences (Liu et al., 2014). Of the labeled ingredients in the Wuhu San prescription, *Angelica dahurica*, *Angelica sinensis,* and *Saposhnikovia divaricata* were all Apiaceae plants. In addition to a small base difference and insufficient assembly accuracy, the low discriminating power of the *rbcL* and *matK* sequences for Apiaceae may also be one reason for the failure to detect *Angelica dahurica* in Wuhu San samples based on these two regions.

The analytical results of this study indicated that the ITS2 sequences displayed the strongest species discriminating power, while that of *psbA-trnH* sequences was lower than the ITS2 sequences. The *matK* and *rbcL* sequences demonstrated the worst species discrimination. Although the discriminating efficiency of the chloroplast *psbA-trnH*, *matK,* and *rbcL* sequences in this study was lower than that of the nuclear ITS2 sequences, the sequences of the chloroplast genome also exhibited certain advantages. The chloroplast genome is mostly maternally inherited and represents single-copy sequences in plant cells (Chen, 2016; Daniell et al., 2016). Moreover, chloroplast DNA sequences can be used as a versatile tool for plant identification (Nock et al., 2011). Most plants contain a significant number of chloroplasts, making DNA easy to obtain. In addition, this study showed that the species obtained from the chloroplast sequences were relatively simple. The obtained sequences generally represented prescription ingredients or obvious adulterants. A combination of multiple DNA barcodes can improve the resolution and accuracy of species discrimination (Group et al., 2009; Arulandhu et al., 2017). In addition, the ITS2 sequences can also detect fungi, which can be used to monitor the potential risk of the fungal contamination of traditional herbal patent medicine (Sweeney and Dobson, 1998; Guo et al., 2020). Therefore, to take advantage of shotgun metabarcoding, combining multiple barcodes obtained via the technology can increase the reliability and applicability of the experimental results. It helps monitor the quality of traditional herbal patent medicine.

## Data Availability

The high-throughput sequencing datasets presented in this study can be found in the National Center for Biotechnology Information (NCBI) SRA online repository. The accession number of the BioProject is PRJNA663116. The accession numbers of the BioSample specimens are SAMN16124456, SAMN16124457, SAMN16124458, SAMN16124459, and SAMN16124460. And the SRA accession numbers for the above five BioSample specimens are SRR12632599, SRR12632598, SRR12632597, SRR12632596, and SRR12632595, respectively. The DNA barcoding sequences assembled from the Sanger sequencing datasets presented in this study can be found in the NCBI GenBank online repository. The accession numbers for these DNA barcoding sequences are MN727081, MN727076, MT821449, MT821451, MT821450, MT102865, MT994327, MT994328, MT994331, MT994330, MT994329, MN729559, MN729561, MW000338, MW000340, MW000339, MW000341, MN746764, MN746766, MW000334, MW000335, and MW000333.
