# Supplementary material for: The Species Identification in Traditional Herbal Patent Medicine, Wuhu San, Based on Shotgun Metabarcoding
Source: Front Pharmacol. 2021 Feb 16;12:607200. doi: 10.3389/fphar.2021.607200 (PMC7921783; doi:10.3389/fphar.2021.607200)
Supplement: Supplementary file 1 [file datasheet1.docx]

## Supplementary Tables

**Supplementary Table 1.** The Latin names of five medicinal materials and their original species.

| Latin name of medicinal materials | Latin Name of original species^1^ |
| --- | --- |
| Angelicae Sinensis Radix | *Angelica sinensis* (Oliv.) Diels |
| Carthami Flos | *Carthamus tinctorius* L. |
| Saposhnikoviae Radix | *Saposhnikovia divaricata* (Turcz. ex Ledeb.) Schischk. |
| Arisaematis Rhizoma Preparatum | *Arisaema erubescens* (Wall.) Schott |
|  | *Arisaema heterophyllum* Blume |
|  | *Arisaema amurense* Maxim. |
| Angelicae Dahuricae Radix | *Angelica dahurica* (Hoffm.) Benth. & Hook.f. ex Franch. & Sav. |
|  | *Angelica dahurica* var. *formosana* (H.Boissieu) Yen |

^1^The Latin names of the original species come from https://mpns.science.kew.org/mpns-portal/?_ga=1.111763972.1427522246.1459077346

**Supplementary Table 2.** The medicinal materials that were used to produce the mock Wuhu San samples.

| Sample ID | Pinyin Name | Latin Name | Original species | Location |
| --- | --- | --- | --- | --- |
| HSYC2002 | Danggui | Angelicae Sinensis Radix | *Angelica sinensis* (Oliv.) Diels | Chengde, Hebei Province |
| HSYC2005 | Honghua | Carthami Flos | *Carthamus tinctorius* L. | Chengde,Hebei Province |
| HSYC2041 | Fangfeng | Saposhnikoviae Radix | *Saposhnikovia divaricata* (Turcz. ex Ledeb.) Schischk. | Chengde, Hebei Province |
| HSYC2077 | Tiannanxing | Arisaematis Rhizoma | *Arisaema amurense* Maxim. | Chengde, Hebei Province |
| HSYC2040 | Baizhi | Angelicae Dahuricae Radix | *Angelica dahurica* (Hoffm.) Benth. & Hook.f. ex Franch. & Sav. | Chengde, Hebei Province |
| HSYC2025 | Xiyangshen | Panacis Quinquefolii Radix | *Panax quinquefolius* L. | Chengde, Hebei Province |

**Supplementary Table 3.** The DNA extraction quality of the Wuhu San samples.

| Sample ID | DNA concentration（ng/μL） | *A_260_/A_280_* |
| --- | --- | --- |
| HSZY160 | 93.3 | 2.0 |
| HSZY172 | 227.3 | 2.01 |
| WHS001 | 94.5 | 1.9 |
| WHS002 | 168 | 1.9 |
| WHS003 | 137.2 | 1.9 |

**Supplementary Table 4.**The sequencing results of the Wuhu San samples.

| Sample ID | Number of bases | Total reads | The number of enriched reads for each DNA barcode | | | | |
| --- | --- | --- | --- | --- | --- | --- | --- |
|  |  |  | ITS2 | *psbA-trnH* | *matK* | *rbcL* | Total |
| HSZY160 | 8537183400 | 28457278 | 37918 | 268449 | 4326 | 7056 | 356985 |
| HSZY172 | 9001356300 | 30004521 | 41361 | 301154 | 4524 | 9226 | 403687 |
| WHS001 | 6806979600 | 22689932 | 23826 | 164017 | 2296 | 3676 | 220962 |
| WHS002 | 6784331400 | 22614438 | 23824 | 169980 | 2492 | 3762 | 227415 |
| WHS003 | 6009891600 | 20032972 | 11266 | 181027 | 2871 | 4466 | 211964 |

**Supplementary Table 5.** The reads number of the prescription ingredients in the three commercial samples based on the ITS2 sequences.

| Latin name | WHS001 | WHS002 | WHS003 |
| --- | --- | --- | --- |
| *Angelica dahurica* | 0 | 0 | 705 |
| *Angelica sinensis* | 1759 | 1877 | 2136 |
| *Arisaema amurense* | 0 | 0 | 0 |
| *Saposhnikovia divaricata* | 2605 | 2888 | 1731 |
| *Carthamus tinctorius* | 10250 | 10677 | 2040 |

**Supplementary Table 6.** The reads number of the prescription ingredients in the three commercial samples based on the *psbA-trnH* sequences.

| Latin name | WHS001 | WHS002 | WHS003 |
| --- | --- | --- | --- |
| *Angelica dahurica* | 0 | 0 | 91 |
| *Angelica sinensis* | 203 | 273 | 313 |
| *Arisaema amurense* | 0 | 0 | 0 |
| *Saposhnikovia divaricata* | 156 | 134 | 443 |
| *Carthamus tinctorius* | 388 | 449 | 127 |

**Supplementary Table 7.** The reads number of the prescription ingredients in the three commercial samples based on the *matK* sequences.

| Latin name | WHS001 | WHS002 | WHS003 |
| --- | --- | --- | --- |
| *Arisaema amurense* | 0 | 0 | 0 |
| *Carthamus tinctorius* | 397 | 698 | 109 |
| *Apiaceae* | 1173 | 1320 | 2056 |

**Supplementary Table 8.** The reads number of the prescription ingredients in the three commercial samples based on the *rbcL* sequences.

| Latin name | WHS001 | WHS002 | WHS003 |
| --- | --- | --- | --- |
| *Arisaema amurense* | 0 | 0 | 0 |
| *Carthamus tinctorius* | 607 | 582 | 190 |
| *Apiaceae* | 471 | 518 | 1086 |

**Supplementary Table 9.** The reads number of the fungi in the five samples based on the ITS2 sequences.

| Family | Genus | Species | HSZY160 | HSZY172 | WHS001 | WHS002 | WHS003 |
| --- | --- | --- | --- | --- | --- | --- | --- |
| Pleosporaceae | *Alternaria* | *Alternaria alternata* | 45 | 20 | 37 | 34 | 97 |
| Aspergillaceae | *Aspergillus* | *Aspergillus flavus* | 0 | 0 | 0 | 0 | 26 |
| Aspergillaceae | *Aspergillus* | *Aspergillus niger* | 0 | 0 | 0 | 0 | 24 |
| Aspergillaceae | *Aspergillus* | *Aspergillus ruber* | 0 | 0 | 48 | 59 | 52 |
| Aspergillaceae | *Aspergillus* | *Aspergillus sp.* | 6 | 0 | 18 | 14 | 29 |
| Saccotheciaceae | *Aureobasidium* | *Aureobasidium pullulans* | 10 | 0 | 0 | 0 | 14 |
| Sclerotiniaceae | *Botrytis* | *Botrytis cinerea* | 0 | 0 | 0 | 0 | 30 |
| Didymellaceae | *Calophoma* | *Calophoma complanata* | 0 | 0 | 0 | 6 | 0 |
| Ceratobasidiaceae | *Ceratobasidium* | *Ceratobasidium sp.* | 0 | 0 | 0 | 0 | 7 |
| Cladosporiaceae | *Cladosporium* | *Cladosporium cladosporioides* | 0 | 0 | 15 | 0 | 14 |
| Cladosporiaceae | *Cladosporium* | *Cladosporium sp.* | 14 | 14 | 0 | 0 | 43 |
| Cystofilobasidiaceae | *Cystofilobasidium* | *Cystofilobasidium macerans* | 0 | 0 | 0 | 0 | 17 |
| Didymellaceae | *Didymella* | *Didymella bellidis* | 0 | 0 | 0 | 12 | 23 |
| Filobasidiaceae | *Filobasidium* | *Filobasidium magnum* | 0 | 0 | 10 | 0 | 17 |
| Nectriaceae | *Fusarium* | *Fusarium acuminatum* | 10 | 0 | 32 | 52 | 0 |
| Nectriaceae | *Fusarium* | *Fusarium equiseti* | 0 | 0 | 13 | 24 | 6 |
| Nectriaceae | *Fusarium* | *Fusarium redolens* | 25 | 94 | 0 | 0 | 0 |
| Nectriaceae | *Fusarium* | *Fusarium solani* | 8 | 15 | 5 | 7 | 0 |
| Nectriaceae | *Fusarium* | *Fusarium sp.* | 0 | 0 | 0 | 0 | 17 |
| Dipodascaceae | *Geotrichum* | *Geotrichum candidum* | 0 | 0 | 8 | 16 | 6 |
| Saccharomycodaceae | *Hanseniaspora* | *Hanseniaspora sp.* | 0 | 0 | 22 | 30 | 0 |
| Botryosphaeriaceae | *Macrophomina* | *Macrophomina phaseolina* | 182 | 133 | 0 | 0 | 100 |
| Mycosphaerellaceae | *Mycocentrospora* | *Mycocentrospora acerina* | 0 | 0 | 17 | 10 | 6 |
| Rhynchogastremataceae | *Papiliotrema* | *Papiliotrema aurea* | 0 | 0 | 0 | 0 | 6 |
| Phaeosphaeriaceae | *Paraphoma* | *Paraphoma chrysanthemicola* | 0 | 0 | 12 | 11 | 8 |
| Aspergillaceae | *Penicillium* | *Penicillium polonicum* | 0 | 0 | 17 | 15 | 8 |
| Herpotrichiellaceae | *Phialophora* | *Phialophora mustea* | 0 | 0 | 5 | 10 | 0 |
| Peronosporaceae | *Phytophthora* | *Phytophthora sp.* | 59 | 52 | 12 | 16 | 0 |
| Pichiaceae | *Pichia* | *Pichia kudriavzevii* | 0 | 0 | 0 | 0 | 26 |
| Plectosphaerellaceae | *Plectosphaerella* | *Plectosphaerella cucumerina* | 0 | 0 | 0 | 6 | 15 |
| Rhizopodaceae | *Rhizopus* | *Rhizopus oryzae* | 7327 | 8073 | 6354 | 6728 | 3409 |
| Rhizopodaceae | *Rhizopus* | *Rhizopus sp.* | 11 | 0 | 0 | 0 | 0 |
| Phaffomycetaceae | *Wickerhamomyces* | *Wickerhamomyces anomalus* | 0 | 0 | 0 | 0 | 24 |
| other fungi | *——* | *Uncultured fungus* | 0 | 0 | 0 | 8 | 0 |
| other fungi | *——* | *Uncultured fungus* | 0 | 0 | 0 | 8 | 0 |
| other fungi | *——* | *Uncultured fungus* | 0 | 8 | 0 | 0 | 0 |

**Supplementary Table 10.** The unique fungi shared at the genus level by the five samples.

| The number of samples | Genera | total |
| --- | --- | --- |
| HSZY160,HSZY172,WHS001,WHS002,WHS003 | *Rhizopus, Alternaria, Fusarium* | 3 |
| HSZY160,HSZY172,WHS001,WHS002 | *Phytophthora* | 1 |
| HSZY160,HSZY172,WHS001,WHS003 | *Cladosporium* | 1 |
| HSZY160,WHS001,WHS002,WHS003 | *Aspergillus* | 1 |
| HSZY160,HSZY172,WHS003 | *Macrophomina* | 1 |
| WHS001,WHS002,WHS003 | *Paraphoma, Mycocentrospora, Geotrichum, Penicillium* | 4 |
| HSZY160,WHS003 | *Aureobasidium* | 1 |
| WHS001,WHS002 | *Phialophora,Hanseniaspora* | 2 |
| WHS001,WHS003 | *Filobasidium* | 1 |
| WHS002,WHS003 | *Plectosphaerella,Didymella* | 2 |
| WHS002 | *Calophoma* | 1 |
| WHS003 | *Wickerhamomyces, Botrytis, Pichia, Papiliotrema, Cystofilobasidium, Ceratobasidium* | 6 |
