# Supplementary figures and images for: The Species Identification in Traditional Herbal Patent Medicine, Wuhu San, Based on Shotgun Metabarcoding
Source: Front Pharmacol. 2021 Feb 16;12:607200. doi: 10.3389/fphar.2021.607200 (PMC7921783; doi:10.3389/fphar.2021.607200)

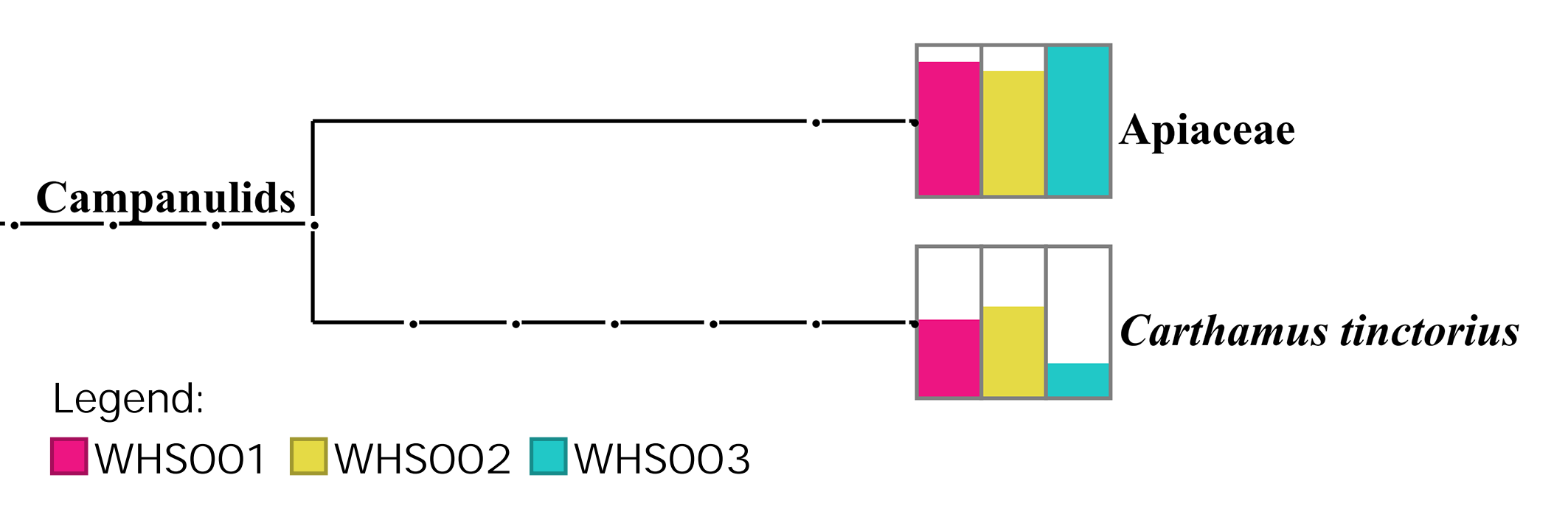

Supplement: Supplementary file 2 [file image1.tif]

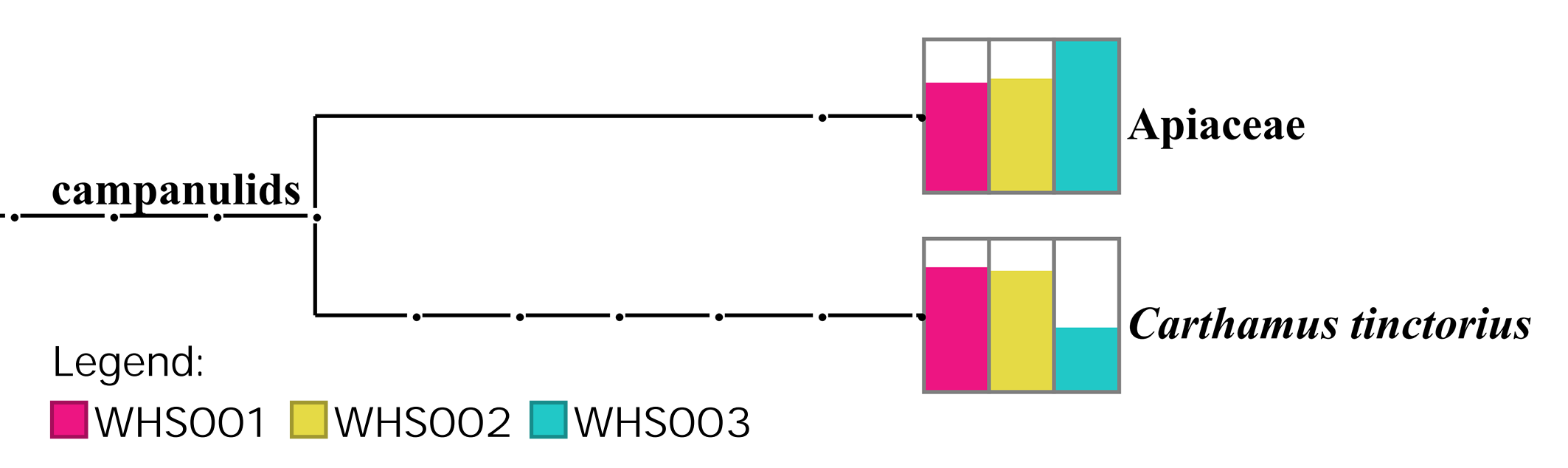

Supplement: Supplementary file 3 [file image2.tif]

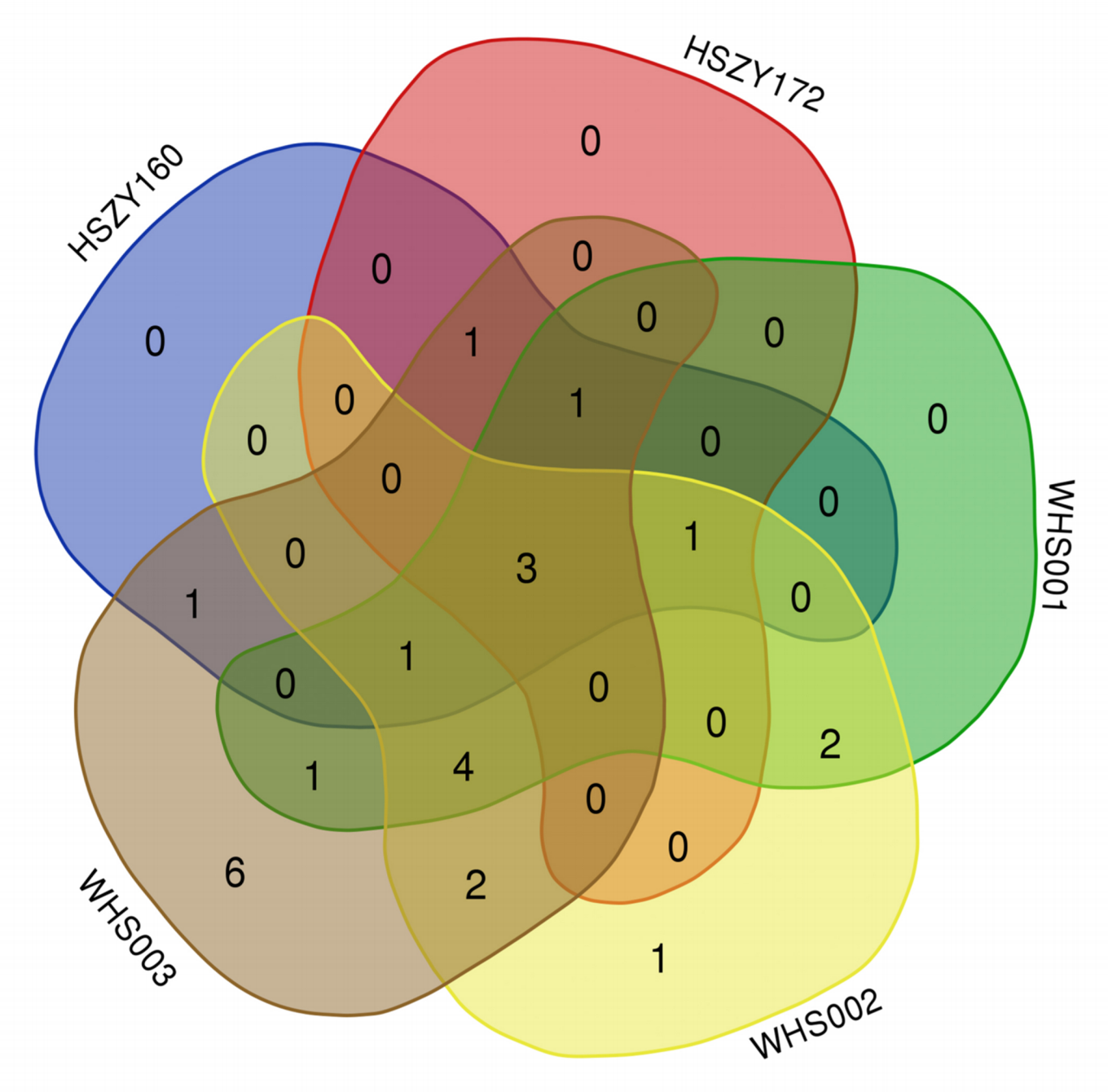

Supplement: Supplementary file 4 [file image3.tif]

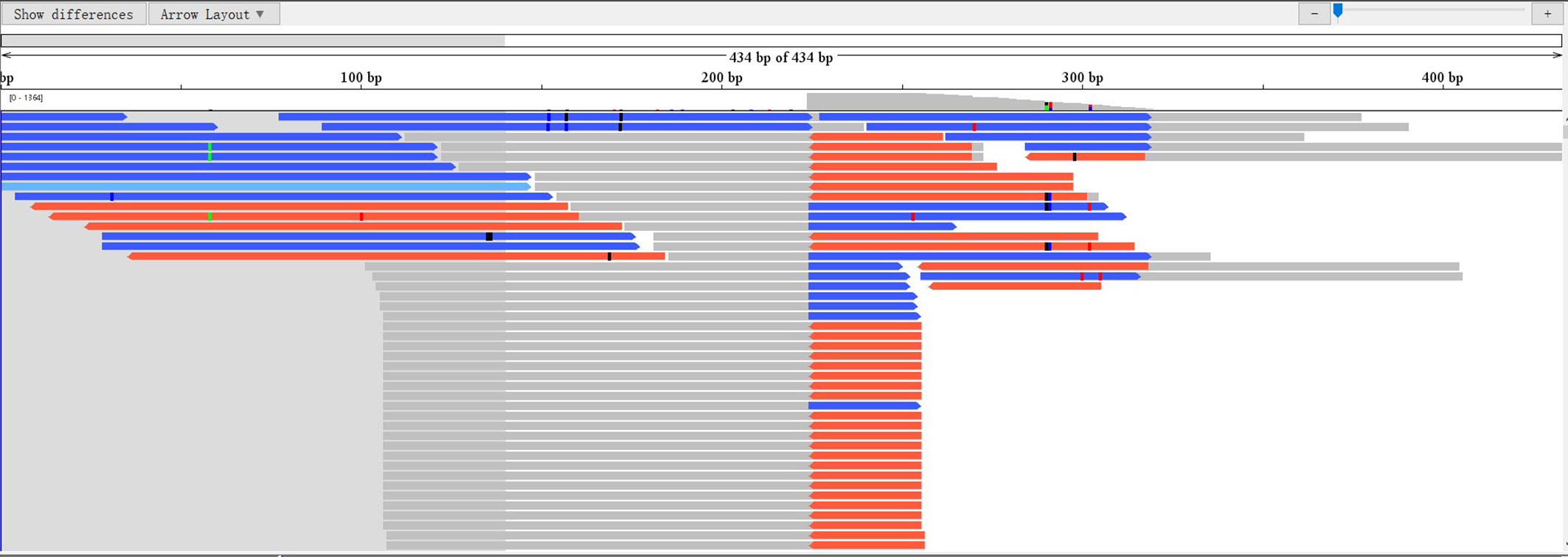

Supplement: Supplementary file 5 [file image4.tif]

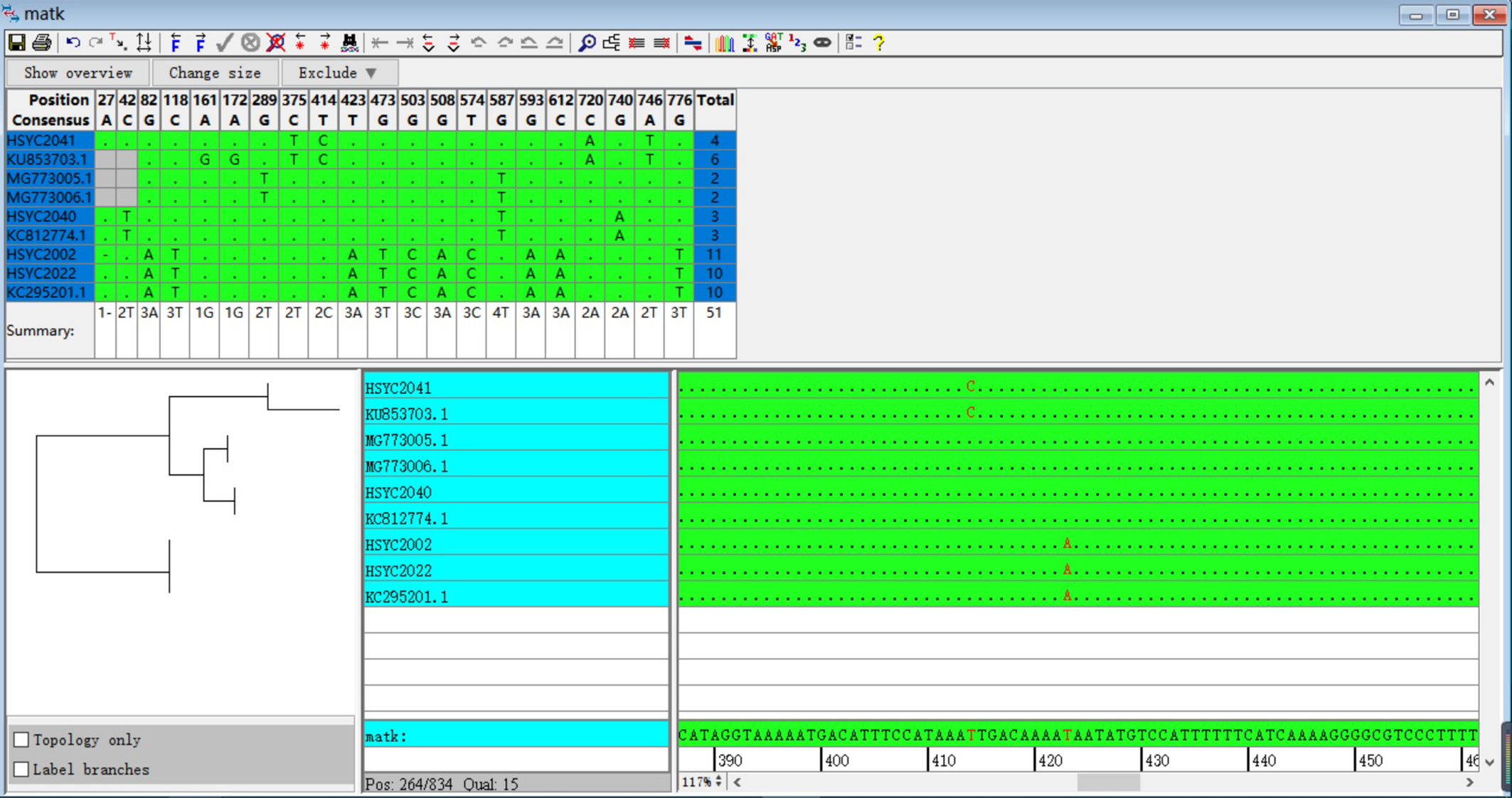

Supplement: Supplementary file 6 [file image5.tif]

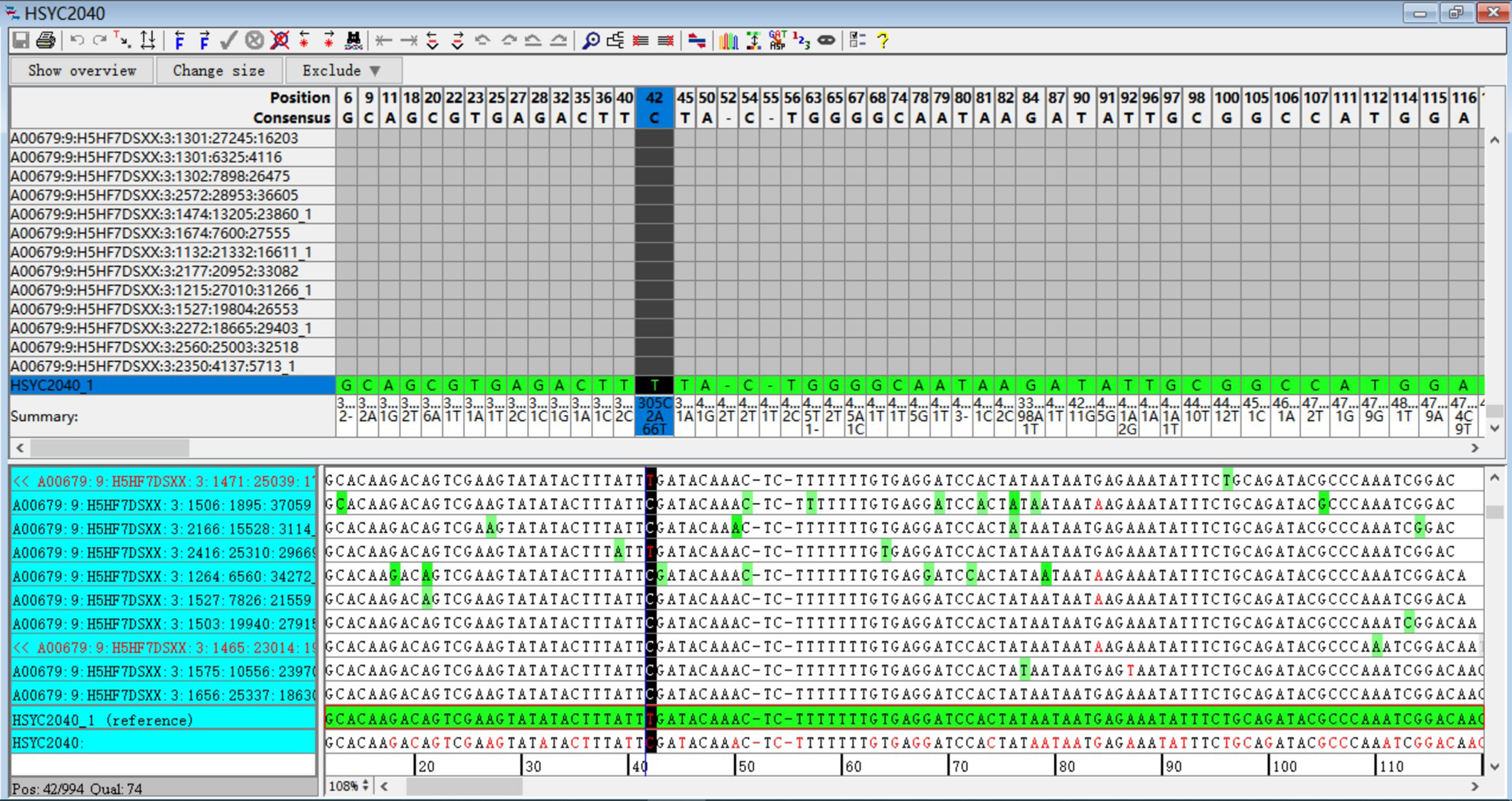

Supplement: Supplementary file 7 [file image6.tif]

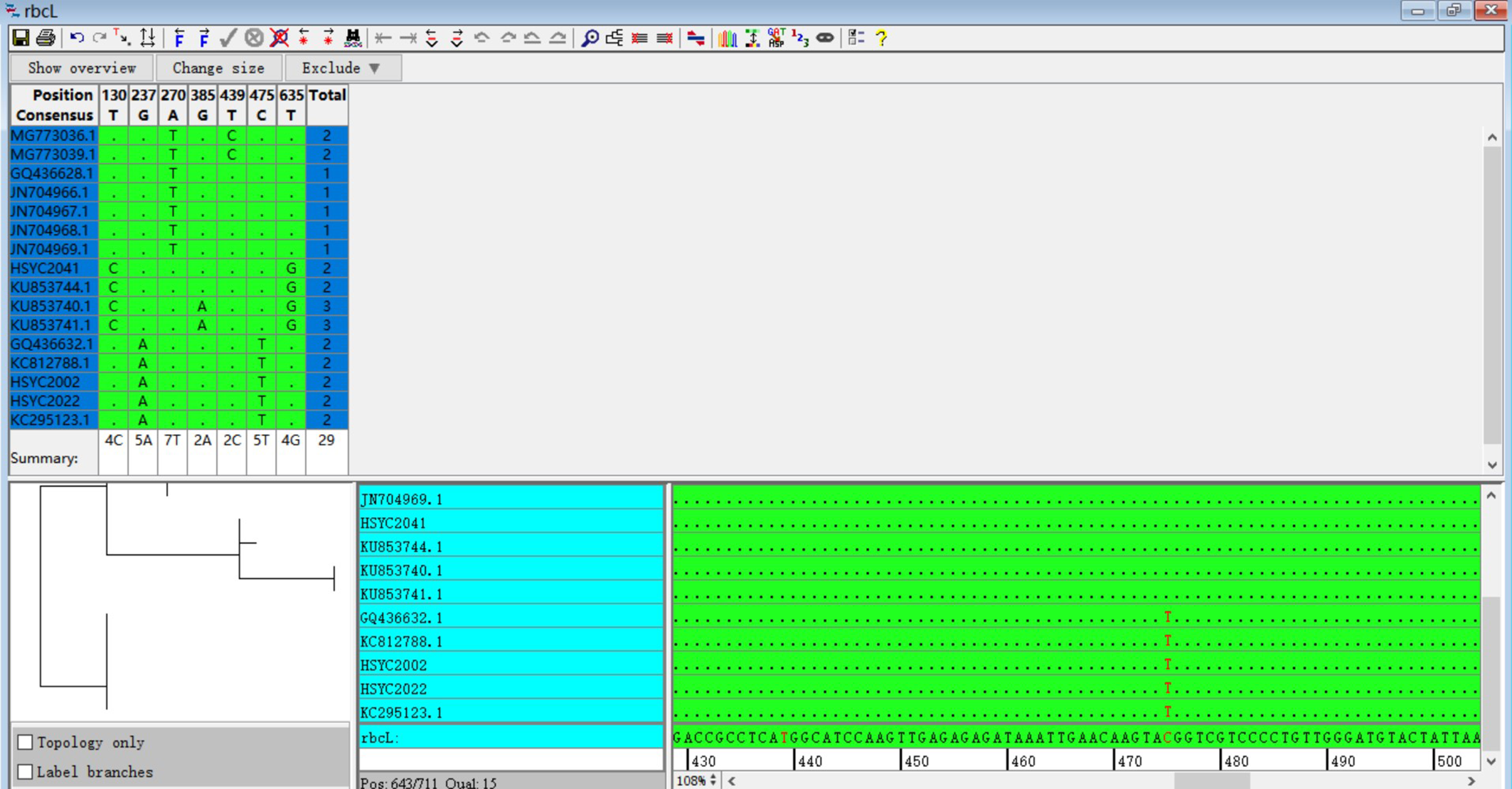

Supplement: Supplementary file 8 [file image7.tif]

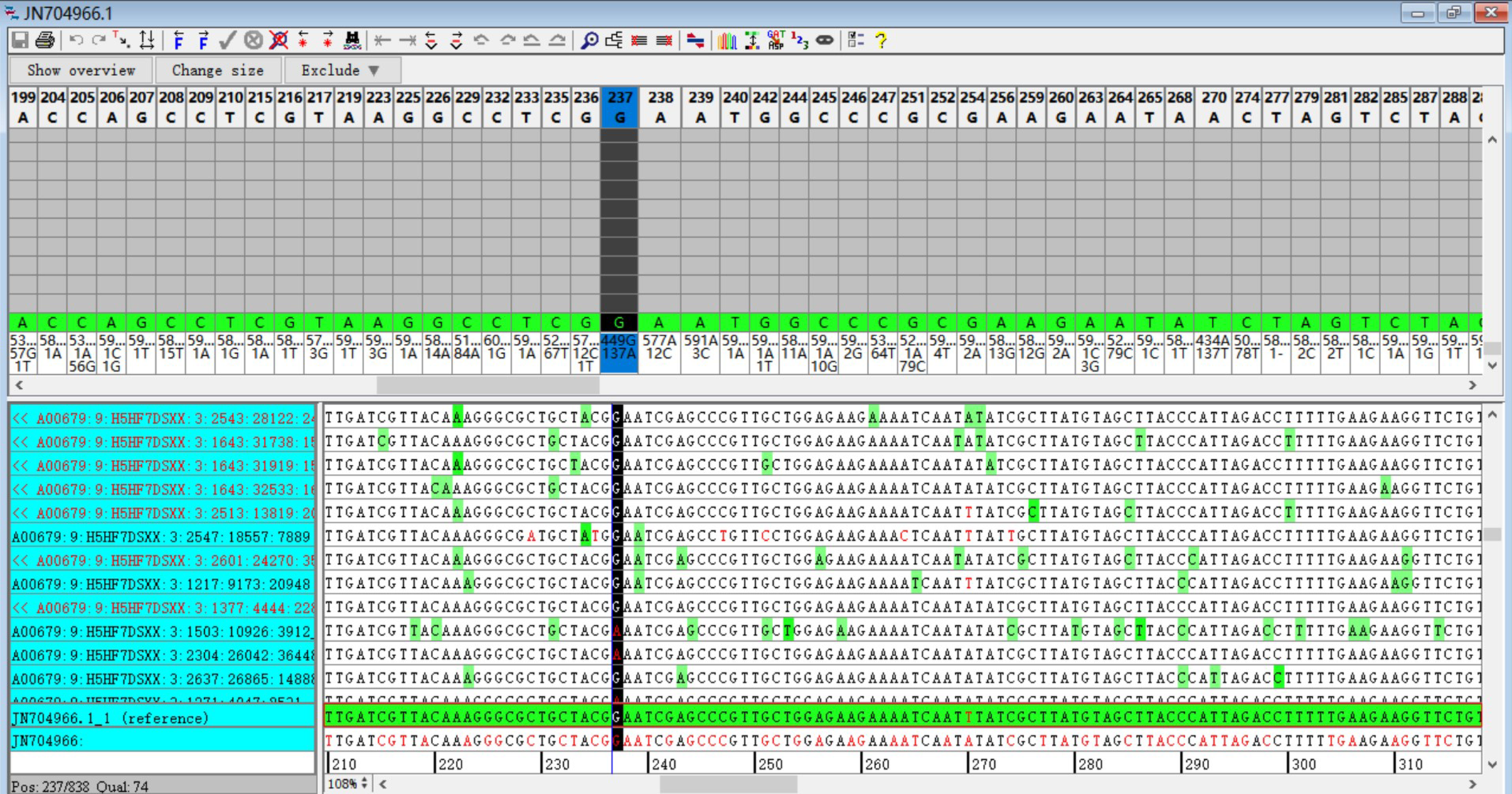

Supplement: Supplementary file 9 [file image8.tif]

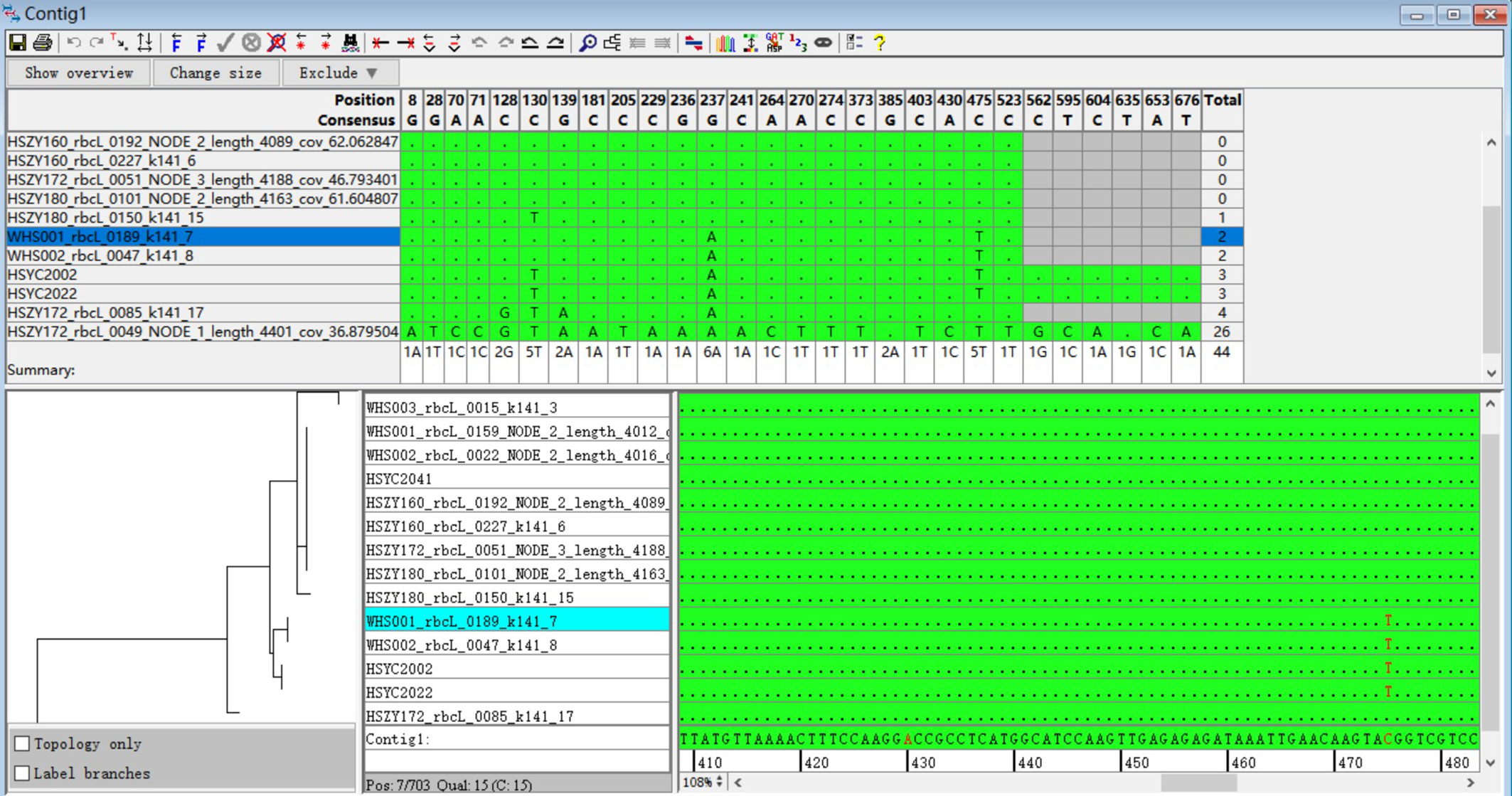

Supplement: Supplementary file 10 [file image9.tif]
